# Overlapping syndrome mimicking infectious meningoencephalitis in a patient with MOG and GFAP IgG

**DOI:** 10.1186/s12883-021-02381-8

**Published:** 2021-09-10

**Authors:** Suqiong Ji, Chenchen Liu, Zhuajin Bi, Huajie Gao, Jian Sun, Bitao Bu

**Affiliations:** Department of Neurology, Tongji Hospital, Tongji Medical College, Huazhong University of Science and Technology, 430030 Wuhan, China

**Keywords:** Overlapping syndrome, Meningoencephalitis, Myelin oligodendrocyte glycoprotein, Glial fibrillary acidic protein

## Abstract

**Background:**

Central nervous system overlapping autoimmune syndromes are uncommon, especially with the coexistence of MOG-IgG and GFAP-IgG.

**Case presentation:**

A 23-year-old woman presented with transient convulsions, a loss of consciousness, persistent fever, headache, and vomiting. Cerebrospinal fluid (CSF) analysis revealed elevated cellularity, and magnetic resonance imaging (MRI) showed diffuse leptomeningeal enhancement. She had fever and headache with antiviral and antibiotic treatment for 2 weeks, and she had empirical anti-tuberculosis treatment and oral prednisolone therapy. She was followed for 3 months after presentation with improved symptoms and normal CSF analysis. A 3-month follow-up MRI showed asymmetric lesions in the cerebellum, corona radiata, and white matter with enhancement. The anti-tuberculosis treatment was continued, and steroid therapy was discontinued. After she stopped taking prednisolone, an interrupted headache gradually appeared. MRI at 4 months after presentation revealed a partial reduction in lesions but enlarged areas in the left cerebellum and right parietal white matter and a new lesion in the region of the right ependyma with linear enhancement. Her CSF was positive for anti-myelin oligodendrocyte glycoprotein (MOG) and anti-glial fibrillary acidic protein (GFAP) antibodies using a transfected cell-based assay. She was diagnosed with overlapping syndrome of MOG‑IgG‑associated disease and GFAP astrocytopathy. She received steroid pulse therapy (methylprednisolone, 1 g for 5 days), followed by a gradual tapering of oral prednisolone and the addition of an immunosuppressant (tacrolimus, 3 mg per day). Six months after the initial presentation, she had no symptoms. An MRI showed that the lesions had diminished, and no enhancement was found.

**Conclusions:**

We report a case that was positive for double antibodies, which was initially misdiagnosed as infectious meningoencephalitis. This case broadens the clinical and phenotypic presentation of the overlapping syndrome spectrum.

## Background

Autoimmune glial fibrillary acidic protein (GFAP) astrocytopathy is a severe inflammatory central nervous system (CNS) disorder that mainly affects the meninges, brain, spinal cord, and optic nerve [1]. GFAP-IgG has been identified as a specific biomarker in serum or cerebrospinal fluid (CSF). Previous studies have found that GFAP-IgG is accompanied by coexisting aquaporin-4 [AQP4 -IgG, N-Methyl-D-aspartate receptor (NMDAR)-IgG, or both] [1–3]. However, the coexistence of GFAP-IgG and myelin oligodendrocyte glycoprotein (MOG)-IgG has rarely been reported. We report a case of overlapping syndrome with the coexistence of MOG-IgG and GFAP-IgG, presenting as clinical meningoencephalitis.

## Case presentation

**Fig. 1:**
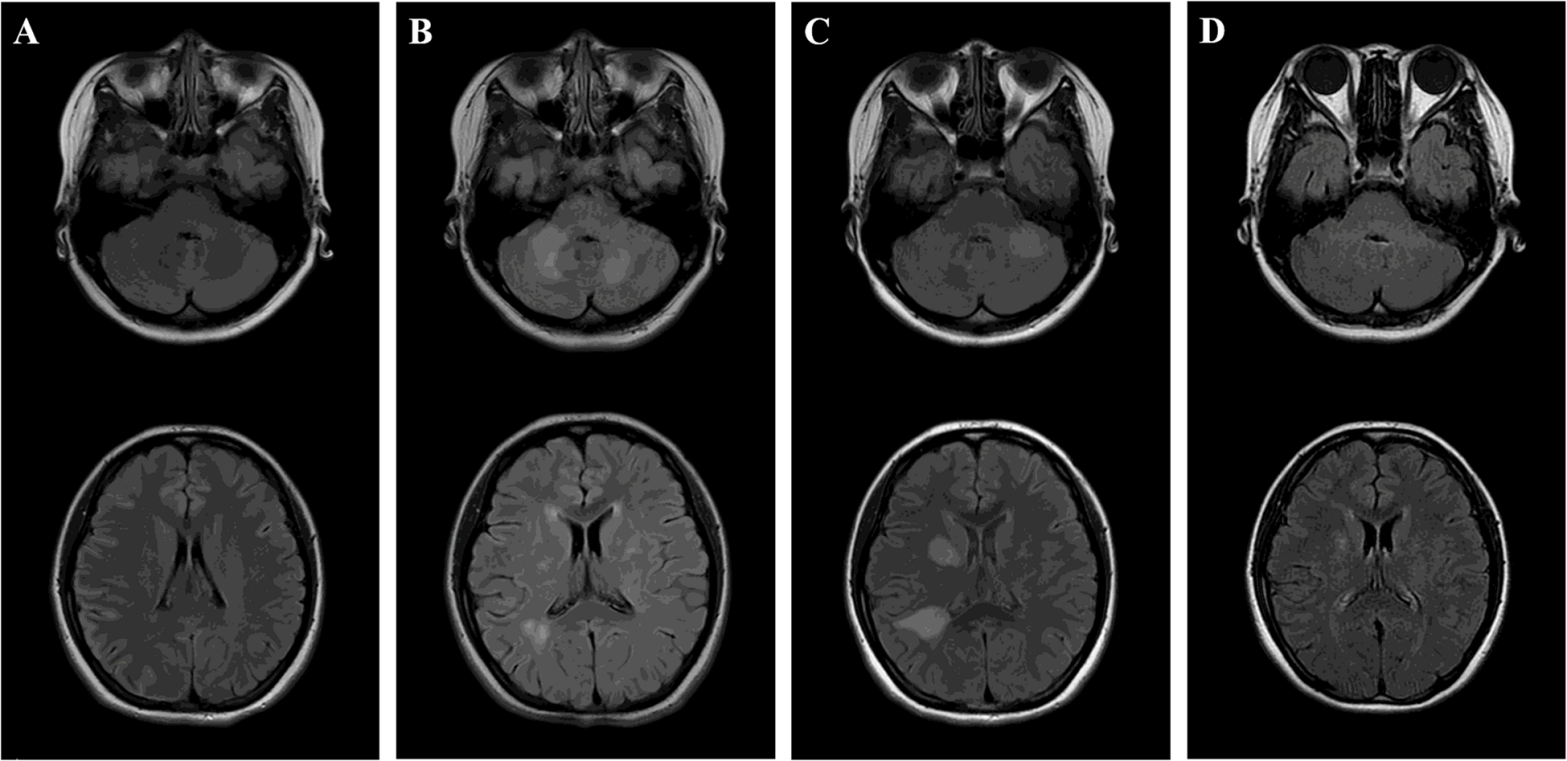
Serial axial T2 FLAIR images at presentation and follow-up. Initial axial brain T2 FLAIR image (**A**) revealed no obvious abnormalities in brain parenchyma. Repeated MRI after 3 months (**B**) showed asymmetric hyperintense signal change of the cerebellum, corona radiata, frontal and parietal white matter. MRI at 4 months from presentation (**C**) showed a partial reduction in lesions, but enlarged areas in left cerebellum and right parietal white matter, and new lesion in the region of the right ependyma. MRI at 6 months from presentation (**D**) revealed obviously resolution of abnormalities

**Fig. 2:**
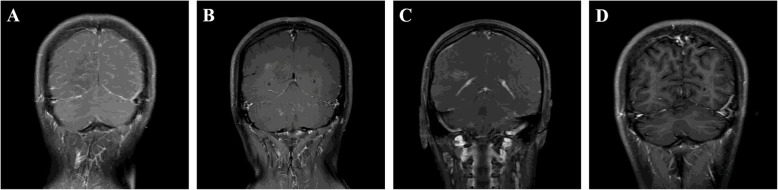
Serial coronal T1 postcontrast MRI at presentation and follow-up. Coronal contrast-enhanced MRI (**A**) revealed diffuse leptomeningeal enhancement at presentation. Repeated enhanced-MRI after 3 months (**B**) showed enhanced lesions in the cerebellum, corona radiata, frontal and parietal white matter. MRI at 4 months from presentation (**C**) showed linearly vessel enhancement in the region of the ependyma. No enhancement was found on enhanced-MRI at 6 months from presentation (**D**)

A 23-year-old woman presented with transient convulsions and a loss of consciousness. She reported a 15-day history of persistent fever, headache, and vomiting without any preceding infection. On admission, a neurological examination was unremarkable except for a positive Kernig sign. CSF analysis revealed elevated cellularity (white blood cell count 210/µL; 60 % lymphocytes, 25 % neutrophils, and 14 % monocytes), a protein level of 537 mg/L, as well as a normal glucose level, cultures for bacteria, tuberculosis, and fungi. Magnetic resonance imaging (MRI) showed no obvious abnormalities in the brain parenchyma (Fig. 1A), but diffuse leptomeningeal enhancement (Fig. 2A). She had fever and headache with antiviral and antibiotic treatment for 2 weeks. Repeat CSF tests still showed leukocytosis (165/µL) and a slightly elevated protein level (554 mg/L). Although there was no laboratory-confirmed diagnosis of tuberculous meningoencephalitis, she was treated with empirical anti-tuberculosis treatment and oral prednisolone therapy. Two weeks later, the fever improved, but the headache persisted. The white blood cell count in the CSF decreased to 80/µL, and CSF protein levels returned to normal.

**Fig. 3:**
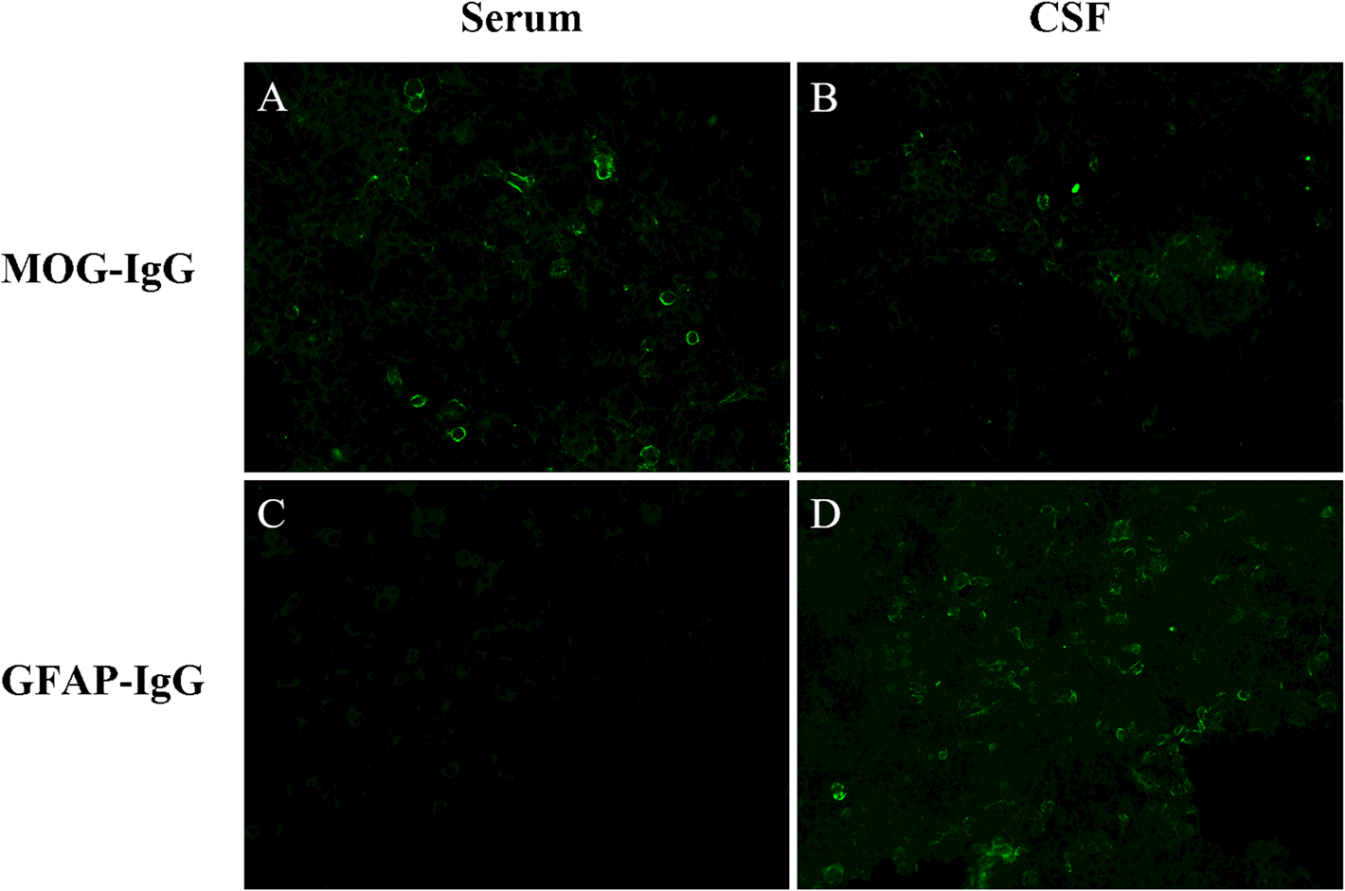
Positive MOG-IgG in serum (**A**) and CSF (**B**), as well as negative GFAP-IgG in serum (**C**) and positive in CSF (**D**) by transfected cell-based assay

She was followed up 3 months after presentation with improved symptoms and normal CSF analysis. However, a follow-up MRI showed asymmetric lesions in the cerebellum, corona radiata, and white matter with enhancement (Figs. 1B and 2B). Anti-tuberculosis treatment was continued, and the steroid was discontinued. After she stopped taking prednisolone, an interrupted headache gradually appeared. The results of a third MRI 4 months after presentation show a partial reduction in lesions, but enlarged areas in the left cerebellum and right parietal white matter, and new lesion in the region of the right ependyma with linear enhancement (Figs. 1C and 2C). Because the anti-infective treatments were ineffective and the characteristic changes on neuroimaging, such as radial enhancement patterns extending outward from the ventricles on enhanced MRI, CNS autoimmune disease was considered as a possible diagnosis. Her CSF was positive for anti-MOG and anti-GFAP antibodies using a transfected cell-based assay, as well as positive anti-MOG antibody and negative anti-GFAP antibody in serum (Fig. 3). Other antibodies against GAD65, NMDAR, GABABR, IgLON5, AMPAR2, DPPX, LGI1, CASPR2, and AQP4 were negative. Spinal and optic nerve MRI revealed no abnormal findings. There was no evidence of neoplasm or paraneoplastic syndrome. She was diagnosed with an overlapping syndrome of MOG‑IgG‑associated disease and GFAP astrocytopathy. She received steroid pulse therapy (methylprednisolone, 1 g for 5 days) followed by a gradual tapering of oral prednisolone and the addition of immunosuppressant (tacrolimus, 3 mg per day). Six months after the patient’s initial presentation, no symptoms were found. An MRI showed that the lesions had diminished (Fig. 1D), and no enhancement was found (Fig. 2D).

## Discussion and conclusion

GFAP astrocytopathy is an immune-mediated inflammatory disease of the CNS. The common clinical features are encephalopathy, papillitis, psychiatric symptoms, seizures, meningeal, and myelopathic symptoms [4]. The CSF demonstrates inflammatory changes in almost all patients. In addition, oligoclonal bands and elevated IgG index can appear in half of the patients [5]. Brain MRI generally shows a characteristic pattern of radial perivascular enhancement through the cerebral white matter emanating from the GFAP-enriched peri-lateral ventricular region [5]. MOG is expressed in the outer lamella of the myelin sheath. Antibodies against this protein are associated with a range of phenotypic presentations, including acute disseminated encephalomyelitis (ADEM), optic neuritis, neuromyelitis optica spectrum disorder (NMOSD), and transverse myelitis [6]. The coexistence of AQP4 or NMDAR antibodies in GFAP astrocytopathy is most common in overlapping syndromes [3]. To our knowledge, only two cases with the coexistence of GFAP-IgG and MOG-IgG have been reported to date, which presented as brainstem encephalitis and optic neuritis [2, 7].

In the present study, we report a case of overlapping syndrome with the coexistence of MOGIgG and GFAP-IgG, misdiagnosed as infectious meningoencephalitis. The overlapping autoimmune syndrome can be a diagnostic challenge in our case, especially on admission. Our patient had persistent fever and headache during the illness, and inflammatory changes were observed in the CSF. However, the absence of abnormalities in the brain parenchyma at presentation could dismiss the diagnosis of autoimmune diseases. GFAP astrocytopathy can also manifest as fever, headache, and seizure, as well as lymphocytic pleocytosis in the CSF. Although the most frequent imaging finding is a striking pattern of linear perivascular radial enhancement on T1-weight post-gadolinium sequence, normal neuroimaging or leptomeningeal enhancement also occurs in more than 20 % of patients with GFAP astrocytopathy [1, 5]. Unfortunately, an anti-GFAP antibody screening was not performed during the first admission in our case, which resulted in a false diagnosis of infectious meningoencephalitis.

GFAP and MOG are expressed in different glial cell types, and different immune mechanisms are shared in GFAP astrocytopathy and MOG-IgG-associated diseases. GFAP, the main intermediate filament protein in astrocytes, is involved in multiple astrocyte functions. Activated cytotoxic T cells may trigger GFAP autoimmunity. Immune-mediated astrocyte dysfunction can lead to chemokine release and further activate the immune response induced by GFAP-specific cytotoxic CD8^+^ T cells, resulting in GFAP astrocytopathy [8]. In addition, GFAP autoimmunity may also occur as a secondary phenomenon. A study from the Mayo Clinic revealed that nearly 40 % of patients with GFAP astrocytopathy had prodromic infectious symptoms [5]. A case report from China also confirmed autoimmune GFAP astrocytopathy after herpes simplex viral encephalitis [9].

In contrast to the mechanism of GFAP astrocytopathy, the production of MOG-IgG is thought to be related to CD4^+^ T cells [10]. MOG-specific B cells are also involved in the activation of MOG-specific T cells and demyelination [11]. However, the underlying mechanisms for patients with the coexistence of GFAP-IgG and MOG-IgG remain elusive. Elevated CSF cytokines, especially interleukin-6, contribute to the pathogenesis of GFAP astrocytopathy and MOG-IgG-positive disorders [12, 13]. Thus, this common pathway in two different pathological mechanisms may play an important role in overlapping syndromes. In addition, increased blood-brain barrier permeability caused by GFAP astrocytopathy can help MOG-IgG enter the central nervous system, resulting in MOG-IgG-associated diseases [14]. In our case, the patient first developed fever and headache, elevated cellularity, protein levels in the CSF, and diffuse leptomeningeal lesions, which are the common manifestations of GFAP astrocytopathy. However, in the next attack, hyperintense signal changes in the cerebellum, corona radiata, and frontal and parietal white matter, which are the common imaging characteristics of MOGIgGmediated disease. Based on the positive serum and CSF MOG-IgG, the patient was diagnosed with MOG-IgG-mediated disease. In addition, linear perivascular radial gadolinium enhancement perpendicular to the ventricle was also observed during the second attack. Taken together, we hypothesized that both MOG-IgG and GFAP-IgG were also involved in the pathogenesis of the second attack. Therefore, we believe that overlapping syndromes of GFAP astrocytopathy and MOG-IgG-associated disease occurred in this patient.

In published cases, GFAP astrocytopathy and MOG-IgG-associated diseases are both sensitive to steroid treatment [1, 15]. Our patient also showed a good response to steroid therapy. Initially, anti-infective treatment was ineffective because of the misdiagnosis of infectious meningoencephalitis, and new lesions gradually appeared in the follow-up MRI. After steroid pulse therapy, the symptoms and imaging findings improved. In addition, a slow steroid taper with close monitoring and long-term immunosuppressive treatment is recommended [16, 17]. With prednisolone and tacrolimus, our patient achieved complete recovery without relapse.

In conclusion, we report a case of overlapping syndrome with the coexistence of MOG-IgG and GFAP-IgG presenting as clinical meningoencephalitis. This report enriches the literature on overlapping autoimmune syndromes. The early screening of autoantibodies against CNS antigens is of great importance for patients suspected of having an intracranial infection for a definite diagnosis. Early steroid treatment and long-term immunosuppression may be appropriate for this rare disease.

## Data Availability

Data sharing is not applicable to this article as no datasets were generated or analysed during the current study.

## Abbreviations

MOG-IgG: Myelin oligodendrocyte glycoprotein -IgG
GFAP-IgG: Glial fibrillary acidic protein-IgG
